# The association between childhood exposure to inter-parental violence and depression in middle-aged and older adults: an empirical analysis from China

**DOI:** 10.1186/s12888-026-08514-y

**Published:** 2026-08-13

**Authors:** Dongxu Li, Wenhui Li, Weile Zhang, Tianjiao Zhang, Zelin Liu, Min Su, Xi Guo

**Affiliations:** 1 School of Public Health, Jining Medical University, Jining, China; 2 School of Public Management, Inner Mongolia University, Hohhot, China; 3 School of Economics and Management, Inner Mongolia University of Technology, Hohhot, China

**Keywords:** Inter-parental violence, Depression, Mediating role, Middle-aged and older adults

## Abstract

**Background:**

The association between exposure to inter-parental violence (IPV) and depression remains to be further explored. This study aimed to explore the association between childhood exposure to IPV and depression in middle-aged and older adults, and to explore the mediating roles between the two.

**Methods:**

Using data from the 2011, 2013, 2015, 2018, and 2020 China Health and Retirement Longitudinal Study (CHARLS), and the 2014 Life History Survey Questionnaire. Generalized estimating equation was used to investigate the relationship between childhood exposure to IPV and depression in middle-aged and older adults, the bootstrap method was used to analyse the mediating roles between the two, and multi-group analysis was used to examine the gender differences in their relationship.

**Results:**

A total of 9,909 sample were included in the study. This study found 18.6% of the sample exposure to IPV in childhood. IPV was positively associated with depression (β = 0.804,95%CI: 0.592,1.015), father-to-mother violence was positively associated with depression (β = 0.777,95%CI: 0.564,0.991), mother-to-father violence was positively associated with depression (β = 0.796,95%CI: 0.434,1.157), and father-and-mother violence was positively associated with depression (β = 0.731,95%CI: 0.351,1.112). IPV and depression may operate partly through smoking and drinking. The association between IPV and depression (*P* = 0.017), and between father-to-mother violence and depression (*P* = 0.023), were both weaker among males than among females.

**Conclusion:**

Childhood exposure to IPV was positively associated with depression in middle-aged and older adults. The association between IPV and depression may operate partly through smoking and drinking. The associations between IPV and depression, and between father-to-mother violence and depression, were stronger among female respondents. There is a need to pay attention to depression among those who exposure to IPV in childhood.

## Introduction

Inter-parental violence (IPV) is a prevalent public health concern [1–3], affecting roughly 30% of families [4–6]. Children exposed to IPV may develop severe emotional disorders [7], and IPV exposure was significantly associated with poorer mental health in adolescents [8].

Numerous studies have examined the association between childhood IPV exposure and depression. Research has identified a significant correlation between IPV exposure and negative emotions in adolescents [9]. Young people exposed to IPV report higher levels of depressive symptoms [10], and they were expected to experience roughly twice the rate of depressive symptoms and suicidal behaviour compared to the general population [11]. Moreover, IPV exposure was correlated with long-term depression. Studies indicated that early exposed to IPV has been shown to increase the risk of depression in adolescence [12, 13]. Additionally, research has found a significant association between childhood IPV exposure and depression among college students [14] and adult men [15]. Further evidence highlighted the sustained negative associations between violent upbringing and adult mental health [16]. Previous research has discovered a direct association between IPV and depression that persisted into adulthood [17], more research was needed to determine whether this association persisted into middle-aged and older adulthood.

Numerous studies have investigated the mediating roles between childhood IPV exposure and later health outcomes. Some have examined lifestyle-related factors and found that IPV exposure was significantly associated with sleep problems [18] and risky internet use [19]. IPV harmed children’s social behaviour and was strongly associated with increased negative communication [20]. Other research indicated that individuals with a history of childhood trauma tended to exhibit a range of cumulative unhealthy lifestyles in adulthood, including smoking, insufficient sleep, and social withdrawal [21]. Furthermore, lifestyle factors such as physical activity, sleep, smoking, diet, and social interaction have been linked to depression [22]. Although IPV was correlated with these lifestyle factors, whether lifestyle mediated the relationship between IPV and depression remained to be elucidated.

In summary, while previous studies have examined the association between IPV and depression in childhood, adolescence, and adulthood, more research was needed to determine whether this association persisted into middle-aged and older adulthood. Additionally, the mediating roles connecting childhood IPV exposure to later-life depression remained to be clarified. Consequently, using longitudinal data from a national follow-up study conducted in China, this study aimed to examine the association between childhood IPV exposure and depression in middle-aged and older adults, evaluated the mediating roles underlying this association, and explored gender differences in their relationship.

## Methods

### Data sources

Data for this study were derived from the China Health and Retirement Longitudinal Study (CHARLS). CHARLS was a national representative longitudinal survey of middle-aged and older Chinese adults, covering 28 provinces, autonomous regions, and municipalities, 150 counties, and 450 communities or villages across the country. The sample accurately reflected the socioeconomic traits, health statuses, and living conditions of middle-aged and older adults in China. CHARLS employed a multistage sampling strategy, with probability proportional to size sampling adopted at both the county and village levels.

CHARLS has carried out five regular waves of longitudinal surveys in 2011, 2013, 2015, 2018, and 2020 respectively. Respondents in each wave were aged 45 years and above, which conformed to the age definition of the middle-aged and older study population in this study. In addition, CHARLS conducted a special survey entitled the Life History Survey in 2014. As a supplementary retrospective survey based on the main CHARLS sample, it collected information on respondents’ adverse childhood experiences occurring before or at age 17, which served as the data source for the core variable of childhood exposure to IPV.

**Fig. 1:**
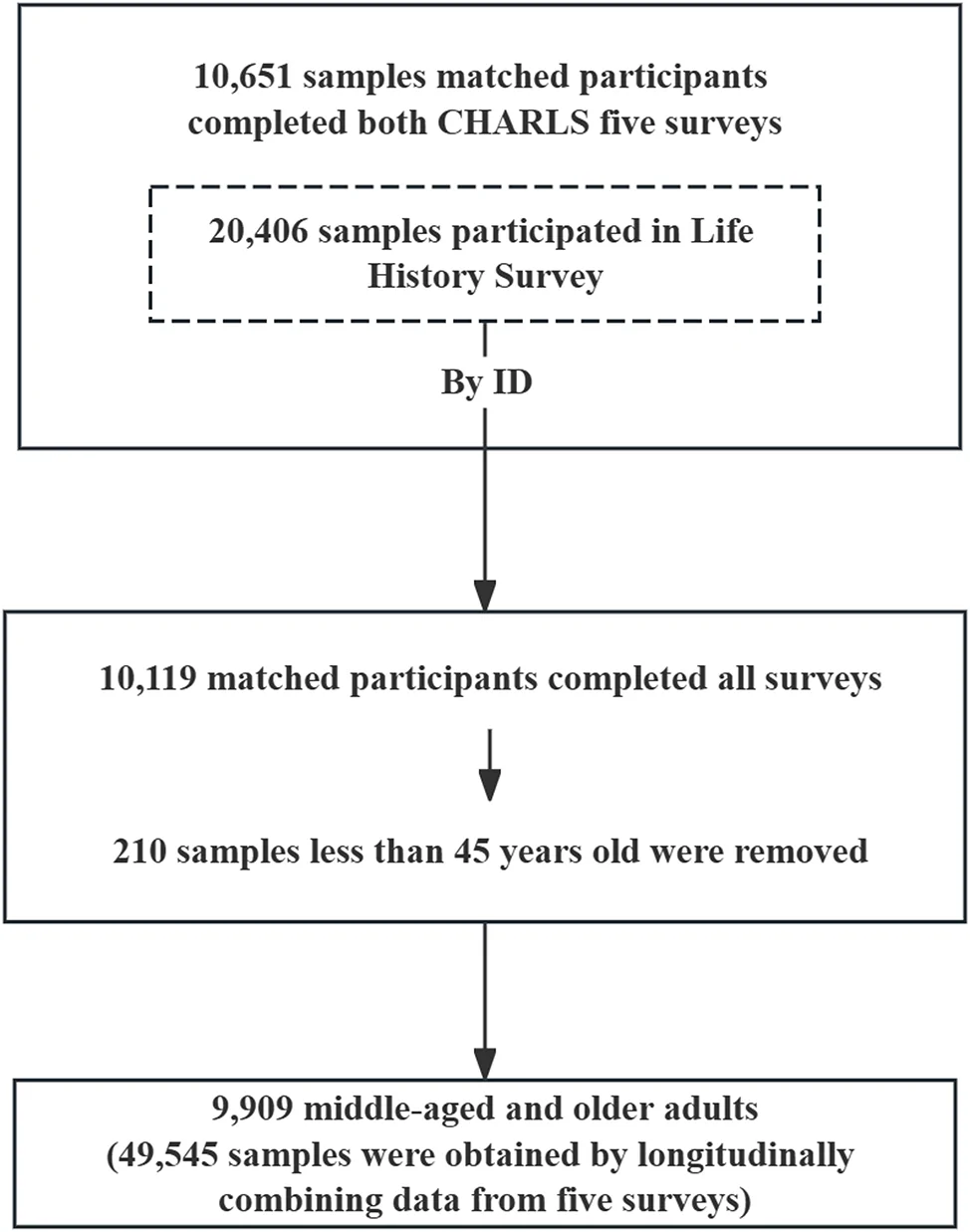
Sample selection flowchart

First, this study screened respondents who participated in all five regular CHARLS waves. Using unique individual ID, the five regular survey datasets were matched with the Life History Survey. This study integrated information on respondents’ depressive symptoms and relevant variables in their middle and old age, along with their childhood exposure to IPV. This process initially yielded 10,119 individuals, from which 210 persons aged under 45 were excluded. After data matching, the final analytic sample comprised 9,909 individuals, contributing a total of 49,545 observations across the five waves. Given the longitudinal design of this study and the sample comprising middle-aged and older adults, variables of gender, age, and educational level completed exhibited stability of variables over time across survey waves. Accordingly, to avoid additional sample attrition, missing values for these variables were supplemented or calculated based on respondent’s baseline survey data. Details are displayed in Fig. 1.

### Variable selection and assignment

#### IPV

Data were obtained from responses to two retrospective questions: ‘Has your father ever beaten your mother?’ and ‘Has your mother ever beaten your father?’. Participants who were exposed to either type of IPV were coded as 1, and those who were not were coded as 0. This study further explored three forms of IPV:

- Participants who solely experienced father-to-mother (F-T-M) violence were coded as 1, and those who did not were coded as 0.
- Participants who solely experienced mother-to-father (M-T-F) violence were coded as 1, and those who did not were coded as 0.
- Participants who were victims of father-and-mother (F&M) violence were coded as 1, and those who were not were coded as 0.

In addition, to facilitate group comparison and robustness testing, this study further defined IPV (four-class): 1 = no exposure to IPV, 2 = only father-to-mother violence, 3 = only mother-to-father violence, 4 = father-and-mother violence.

### Depression

The Center for Epidemiologic Studies Depression Scale-10 (CES-D-10) was used to measure depression. Depression among middle-aged and older adults aged 45 years and above was measured and tracked in each CHARLS wave. The ten questions on the CES-D-10 response scale were about the participant’s experiences over the past week, including feeling troubled, having difficulty concentrating, and more. The Chinese version of the scale, where 0 = 1 day, 1 = 1–2 days, 2 = 3–4 days, and 3 = 5–7 days, was used to ask participants to respond to each question. A total score calculated by adding the scores for each question (the scores of the two positive items on the scale were reversed prior to summation).

With a measured reliability and validity coefficient of 0.8, CES-D-10 has proven to have excellent psychometric properties among middle-aged and older Chinese adults [23, 24]. According to previous research [25, 26], depression had a total score between 0 and 30, with higher scores indicating more severe depressive symptoms.

In addition, according to the previous classification methods [27, 28], for robustness testing, this study further defined binary depression: a CES-D-10 score of less than 10 was classified as indicating no depression, receiving a value of 0. Conversely, a score of 10 or higher was classified as indicating depression and assigned a value of 1.

### Mediating variable

Referring to previous studies [29, 30], this study selected smoking, drinking, social contact, exercising, and sleep duration as mediating variables.

- Smoking was derived from the question “Have you ever chewed tobacco, smoked a pipe, smoked self-rolled cigarettes, or smoked cigarettes/cigars?” Respondents who answered “Yes” were coded as 1, while “No” respondents were coded as 0.
- Drinking was derived from the question “Did you drink any alcoholic beverages, such as beer, wine, or liquor in the past year? How often?” Those who did not drink were coded as 0, those who drank less than once a month as 1, and those who drank more than once a month as 2.
- Social contact was measured by the question “Have you any of social activities in the in the last month?” Respondents who answered “No” were coded as 0, and those who answered “Yes” as 1.
- Exercising was based on the question “During a usual week, did you do any high intensity, moderate, or mild exercising for at least 10 minutes continuously?” Those who reported engaging in exercising were coded as 1, and others as 0.
- Sleep duration was obtained from the question “During the past month, how many hours of actual sleep did you get at night?” This variable was treated as a continuous variable.

### Covariates

Referring to previous research [31, 32], potential confounders included sociodemographic variables: gender (Female = 0, Male = 1), age (Continuous variable), income (Continuous variable), area of residence (Rural = 0, Urban = 1), marital status (Unmarried = 0, Married = 1), educational level completed (Primary school or below = 0, Middle school or above = 1).

Chronic diseases and lifestyle factors were linked with depression, as indicated by previous studies [33, 34]. Therefore, chronic disease was selected as a control variable. Participants were assigned a value of 1 if they had at least one of the 14 chronic conditions listed in the CHARLS database and 0 otherwise.

This study also included childhood confounding variables, encompassing childhood socioeconomic status (Low = 1, Middle = 2, High = 3), child physical abuse (No = 0, Yes = 1), parental alcohol use (No = 0, Yes = 1), parental mental illness (No = 0, Yes = 1), and parental divorce (No = 0, Yes = 1). Additionally, time and region fixed effects were considered in this study.

### Statistical analysis

This study conducted regression analyses using the generalized estimating equation model, with participant ID set as the clustering variable. The model specified an exchangeable working correlation structure, an identity link function, and a Gaussian distribution family, and robust standard errors were utilized for statistical analysis. This study contained 9,909 participants, each completing five rounds of follow-up surveys, yielding a total of 49,545 person-wave observations. Survey wave fixed effects were adjusted in the regression model.

In the baseline regression (Model 1 to Model 4), this study examined the associations of IPV, father-to-mother violence, mother-to-father violence, and father-and-mother violence with depression, respectively.

This study adopted the variable replacement method for robustness tests. Model 5 examined the association between IPV (four-class) and depression, while Model 6 examined the association between IPV and binary depression. If associations remained statistically significant, the results were considered robust.

In the mediation analysis, this study used a lagged mediation model based on bootstrap sampling [35] and bias-corrected 95% confidence interval (CI) to investigate the mediating roles between IPV and depression. The exposure variable was derived from Life History Survey. Mediating variable, covariates, and depression were obtained from the CHARLS 2011–2020, with measurements taken when participants were aged 45 years and older.

For the lagged mediation design, the mediating variable was assessed at wave T, and the covariates and depression were assessed at waves T + 1, T + 2, T + 3, and T + 4. Repeated observations were stacked longitudinally within each lag specification. This yielded lag-1, lag-2, lag-3, and lag-4 mediation models. The Models controlled for baseline depression measured at wave T.

Heterogeneity analysis was performed using multi-group analysis by gender subgroups. The associations of childhood exposure to IPV, father-to-mother violence, mother-to-father violence, and father-and-mother violence with depression in middle-aged and older adults were analyzed separately for males and females, and interaction analysis was further conducted to test for gender differences in these associations.

All data analyses were completed using STATA 15.1. The significance level for all hypothesis tests was set at 0.05.

## Results

### Descriptive statistics

This study involved 9,909 respondents, of which 5,314 (53.6%) were females, and 4,595 (46.4%) were males. The average age was about 58 years, and the average income was about 10,379 Chinese Yuan (1 US dollar = 6.46 Chinese Yuan). Of the respondents, 6,625 (66.9%) were from rural areas, and 3,284 (33.1%) were from urban areas. Among the participants, 8,919 (90.0%) were married. A total of 6,775 individuals (68.4%) had a primary school education or below, while 3,134 (31.6%) had completed middle school or higher. Additionally, 6,632 individuals (66.9%) suffered from chronic diseases. Among the respondents, 4,018 (40.5%) had a low socioeconomic status as children, 5,050 (51.0%) had a middle status, and 841 (8.5%) had a high status. The number of respondents who had experienced physical abuse as children was 5,694 (57.5%), 679 (6.8%) had reported parental alcohol abuse, 351 (3.5%) had reported parental mental illness, and 80 (0.8%) had experienced parental divorce. Among IPV exposure, 8,060 (81.4%) participants had no IPV exposure, and 1,849 (18.6%) had IPV exposure. 1,391(14.0%) were exposed only to father-to-mother violence, 43 (0.4%) were exposed only to mother-to-father violence,415 (4.2%) were exposed to father-and-mother violence. Details are given in Table 1.

**Table 1:** Descriptive statistical analysis (2011, *N* = 9,909)

Variables		Total	
		*N*	%
IPV (four-class)	No exposure to IPV	8,060	81.4
	Only F-T-M violence	1,391	14.0
	Only M-T-F violence	43	0.4
	F&M violence	415	4.2
Gender	Female	5,314	53.6
	Male	4,595	46.4
Age	Mean ± S.D.	58.153 ± 8.632	
Income	Mean ± S.D.	10378.751 ± 31930.036	
Area of residence	Rural	6,625	66.9
	Urban	3,284	33.1
Marital status	Married	8,919	90.0
	Unmarried	990	10.0
Educational level completed	Primary school or below	6,775	68.4
	Middle school or above	3,134	31.6
Chronic disease	No	3,277	33.1
	Yes	6,632	66.9
Childhood socioeconomic status	Low	4,018	40.5
	Middle	5,050	51.0
	High	841	8.5
Child physical abuse	No	4,215	42.5
	Yes	5,694	57.5
Parental alcohol use	No	9,230	93.2
	Yes	679	6.8
Parental mental illness	No	9,558	96.5
	Yes	351	3.5
Parental divorce	No	9,829	99.2
	Yes	80	0.8
*N*		9,909	100

### Benchmark regression: the association between childhood exposure to IPV and depression in middle-aged and older adults

Model 1 assessed the association between childhood exposure to IPV and depression in middle-aged and older adults, the results demonstrated that IPV was positively associated with depression (β = 0.804,95%CI: 0.592,1.015). The results of Model 2 demonstrated that father-to-mother violence was positively associated with depression (β = 0.777,95%CI: 0.564,0.991). Although the sample size of participants exposure to mother-to-father violence was relatively small, the results of Model 3 demonstrated that mother-to-father violence was positively associated with depression (β = 0.796,95%CI: 0.434,1.157). The results of Model 4 demonstrated that father-and-mother violence was positively associated with depression (β = 0.731,95%CI: 0.351,1.112). Details are provided in Table 2.

**Table 2:** Analysis of the association between IPV and depression

Variables	Model 1	Model 2	Model 3	Model 4
	Depression	Depression	Depression	Depression
IPV	0.804^***^			
	[0.592,1.015]			
F-T-M violence		0.777^***^		
		[0.564,0.991]		
M-T-F violence			0.796^***^	
			[0.434,1.157]	
F&M violence				0.731^***^
				[0.351,1.112]
Gender	−1.666^***^	−1.668^***^	−1.670^***^	−1.672^***^
	[− 1.829, − 1.503]	[− 1.832, − 1.505]	[− 1.833, − 1.506]	[− 1.836, − 1.509]
Age	−0.006	−0.006	−0.007	−0.007
	[− 0.016, 0.003]	[− 0.016, 0.003]	[− 0.016,0.003]	[− 0.016,0.003]
Income	−0.060^***^	−0.060^***^	−0.060^***^	−0.060^***^
	[− 0.072, − 0.048]	[− 0.072, − 0.048]	[− 0.072, − 0.047]	[− 0.072, − 0.047]
Area of residence	−1.084^***^	−1.084^***^	−1.104^***^	−1.104^***^
	[− 1.254, − 0.913]	[− 1.255, − 0.914]	[− 1.274, − 0.933]	[− 1.274, − 0.933]
Marital status	−1.093^***^	−1.093^***^	−1.080^***^	−1.081^***^
	[− 1.312, − 0.875]	[− 1.312, − 0.875]	[− 1.299, − 0.861]	[− 1.300, − 0.862]
Educational level completed	−1.085^***^	−1.084^***^	−1.095^***^	−1.094^***^
	[− 1.271, − 0.898]	[− 1.270, − 0.897]	[− 1.282, − 0.908]	[− 1.281, − 0.907]
Chronic disease	1.456^***^	1.458^***^	1.469^***^	1.471^***^
	[1.295, 1.617]	[1.297, 1.619]	[1.308,1.631]	[1.309, 1.633]
Childhood socioeconomic status				
Middle	−0.854^***^	−0.856^***^	−0.870^***^	−0.871^***^
	[− 1.024, − 0.685]	[− 1.025, − 0.687]	[− 1.039, − 0.701]	[− 1.040, − 0.702]
High	−1.242^***^	−1.241^***^	−1.264^***^	−1.263^***^
	[− 1.534, − 0.950]	[− 1.533, − 0.949]	[− 1.556, − 0.972]	[− 1.555, − 0.971]
Child abuse	0.381^***^	0.389^***^	0.482^***^	0.488^***^
	[0.214, 0.548]	[0.222, 0.556]	[0.318,0.646]	[0.324,0.652]
Parental alcohol use	0.216	0.218	0.281	0.282
	[− 0.106,0.537]	[− 0.103,0.540]	[− 0.039,0.600]	[− 0.037,0.602]
Parental mental illness	1.688^***^	1.691^***^	1.728^***^	1.730^***^
	[1.234, 2.142]	[1.236,2.145]	[1.270,2.185]	[1.272,2.189]
Parental divorce	0.986^*^	0.982^*^	0.973^*^	0.970^*^
	[0.048,1.925]	[0.044,1.920]	[0.032,1.915]	[0.028,1.911]
Time effect	Yes	Yes	Yes	Yes
Regional effect	Yes	Yes	Yes	Yes
*Wald chi2*	3146.21	3137.80	3109.18	3100.54
*P*	< 0.001	< 0.001	< 0.001	< 0.001
*N*	49,545	49,545	49,545	49,545

NOTE: 95% CI in parentheses. *P* < 0.05, ** *P* < 0.01, *** *P* < 0.001. Model 1 demonstrated association between childhood exposure to IPV and depression in middle-aged and older adults, Model 2 demonstrated association between father-to-mother violence and depression, Model 3 demonstrated association between mother-to-father violence and depression, Model 4 demonstrated association between father-and-mother violence and depression. The independent variables of Model 1 to Model 4 were IPV, father-to-mother violence, mother-to-father violence, and father-and-mother violence, all of which were dichotomous variables. The dependent variable was depression, treated as a continuous variable

Model 5 treated IPV as a four-class variable, and the results demonstrated that exposure only to father-to-mother violence was positively associated with depression (β = 0.758, 95% CI: 0.518,0.998), exposure only to mother-to-father violence was positively associated with depression (β = 1.473,95%CI: 0.388,2.558), exposure to father-and-mother violence was positively associated with depression (β = 0.888,95%CI: 0.506,1.270). The results of Model 6 demonstrated that IPV was positively associated with binary depression (β = 0.054,95%CI: 0.039, 0.069). Details are given in Table 3.

**Table 3:** Robustness analysis of the association between IPV and depression

Variables	Model 5	Model 6
	Depression	Binary depression
IPV (four-class)		
F-T-M violence	0.758^***^	
	[0.518,0.998]	
M-T-F violence	1.473^*^	
	[0.388,2.558]	
F&M violence	0.888^***^	
	[0.506,1.270]	
IPV		0.054^***^
		[0.039, 0.069]
Gender	−1.664^***^	−0.105^***^
	[− 1.827, − 1.501]	[− 0.117, − 0.093]
Age	−0.006	−0.003^***^
	[− 0.016,0.004]	[− 0.003, − 0.002]
Income	−0.060^***^	−0.005^***^
	[− 0.072, − 0.048]	[− 0.006, − 0.004]
Area of residence	−1.085^***^	−0.065^***^
	[− 1.255, − 0.914]	[− 0.078, − 0.053]
Marital status	−1.092^***^	−0.050^***^
	[− 1.310, − 0.873]	[− 0.066, − 0.034]
Educational level completed	−1.087^***^	−0.047^***^
	[− 1.273, − 0.900]	[− 0.060, − 0.033]
Chronic disease	1.455^***^	0.097^***^
	[1.294,1.616]	[0.085, 0.109]
Childhood socioeconomic status		
Middle	−0.855^***^	−0.051^***^
	[− 1.024, − 0.686]	[− 0.063, − 0.039]
High	−1.244^***^	−0.075^***^
	[− 1.536, − 0.952]	[− 0.096, − 0.055]
Child abuse	0.380^***^	0.035^***^
	[0.213, 0.547]	[0.023, 0.047]
Parental alcohol use	0.217	0.014
	[− 0.104,0.539]	[− 0.009, 0.038]
Parental mental illness	1.687^***^	0.102^***^
	[1.233,2.141]	[0.071, 0.133]
Parental divorce	0.989^*^	0.053
	[0.050,1.927]	[− 0.010,0.116]
Time effect	Yes	Yes
Regional effect	Yes	Yes
*Wald chi2*	3153.96	2209.89
*P*	< 0.001	< 0.001
*N*	49,545	49,545

NOTE: 95% CI in parentheses. *P* < 0.05, ** *P* < 0.01, *** *P* < 0.001. Model 5 demonstrated association between childhood exposure to IPV (four-class) and depression, Model 6 showed association between IPV and binary depression

### Mediating roles of the association between IPV and depression

**Fig. 2:**
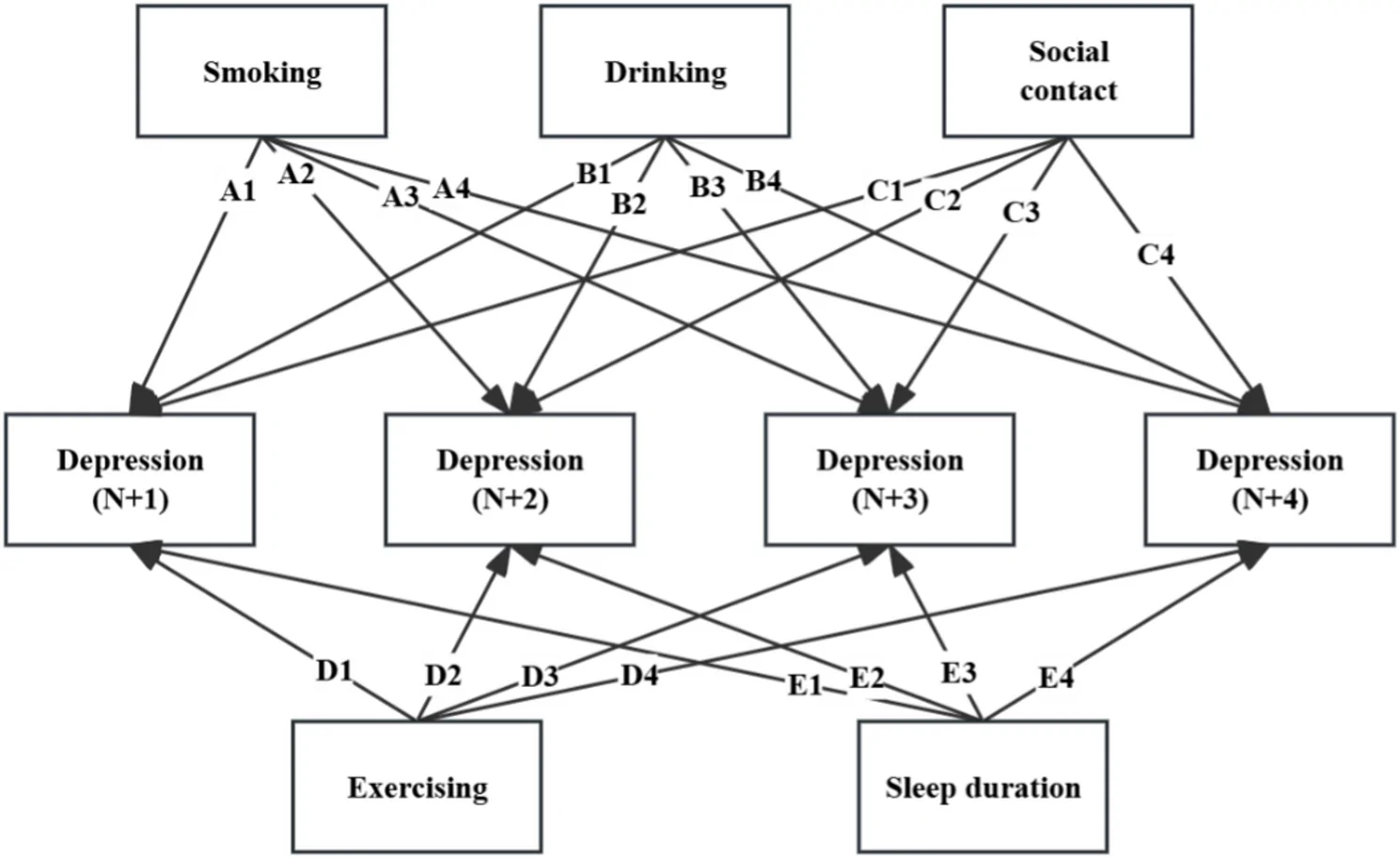
Analysis on the mediating role of the association between IPV and depression. NOTE: Figure 2 showed the indirect effects. The independent variable was IPV (any IPV), the mediators were smoking, drinking, social contact, exercising, and sleep duration, and the dependent variable was depression (1-wave lag, 2-wave lag, 3-wave lag, and 4-wave lag). *N* + 1, *N* + 2, *N* + 3, and *N* + 4 represented depression at the first, second, third, and fourth lagged waves, respectively. Depression was measured as a continuous variable. A1-A4 represented the mediating role of smoking in the association between IPV and depression from the first to the fourth lagged waves, where A1 (β = 0.004,95%CI: 0.002, 0.009), A2 (β = 0.004,95%CI: 0.001, 0.009), A3 (β = 0.002,95%CI: −0.001, 0.007), and A4 (β=−0.001,95%CI: −0.006, 0.002). B1-B4 represented the mediating role of drinking in the association between IPV and depression from the first to the fourth lagged waves, where B1 (β = −0.005,95%CI: −0.009, − 0.002), B2 (β = −0.003,95%CI: −0.007, 0.001), B3 (β = −0.004,95%CI: −0.011, 0.002), and B4 (β= −0.005,95%CI: −0.018, 0.002).C1-C4 represented the mediating role of social contact in the association between IPV and depression from the first to the fourth lagged waves, where C1 (β = −0.003,95%CI: −0.007, 0.001), C2 (β = −0.002,95%CI: −0.006, 0.002), C3 (β = 0.001,95%CI: −0.004, 0.006), and C4 (β = 0.003,95%CI: −0.005, 0.011). D1-D4 represented the mediating role of exercising in the association between IPV and depression from the first to the fourth lagged waves, where D1 (β =−0.001,95%CI: −0.005, 0.002), D2 (β =−0.001,95%CI: −0.004, 0.001), D3 (β =−0.001,95%CI: −0.005, 0.002), and D4 (β=−0.001,95%CI: −0.009, 0.002). E1-E4 represented the mediating role of sleep duration in the association between IPV and depression from the first to the fourth lagged waves, where E1 (β = 0.005,95%CI: −0.005, 0.016), E2 (β = 0.004,95%CI: −0.004, 0.012), E3 (β = 0.006,95%CI: −0.003, 0.016), and E4 (β = 0.005,95%CI: −0.009, 0.001)

The mediating role of smoking, drinking, social contact, exercising, and sleep duration were investigated in this study using lagged mediation analysis. The results demonstrated that the association between IPV and depression at the first (A1, β = 0.004,95%CI: 0.002, 0.009) and second (A2, β = 0.004,95%CI: 0.001, 0.009) lagged waves may operate partly through smoking. The results demonstrated that the association between IPV and depression at the first (B1, β = −0.005,95%CI: −0.009, − 0.002) lagged wave may operate partly through drinking. Details are displayed in Fig. 2.

### Heterogeneity analysis: gender

**Fig. 3:**
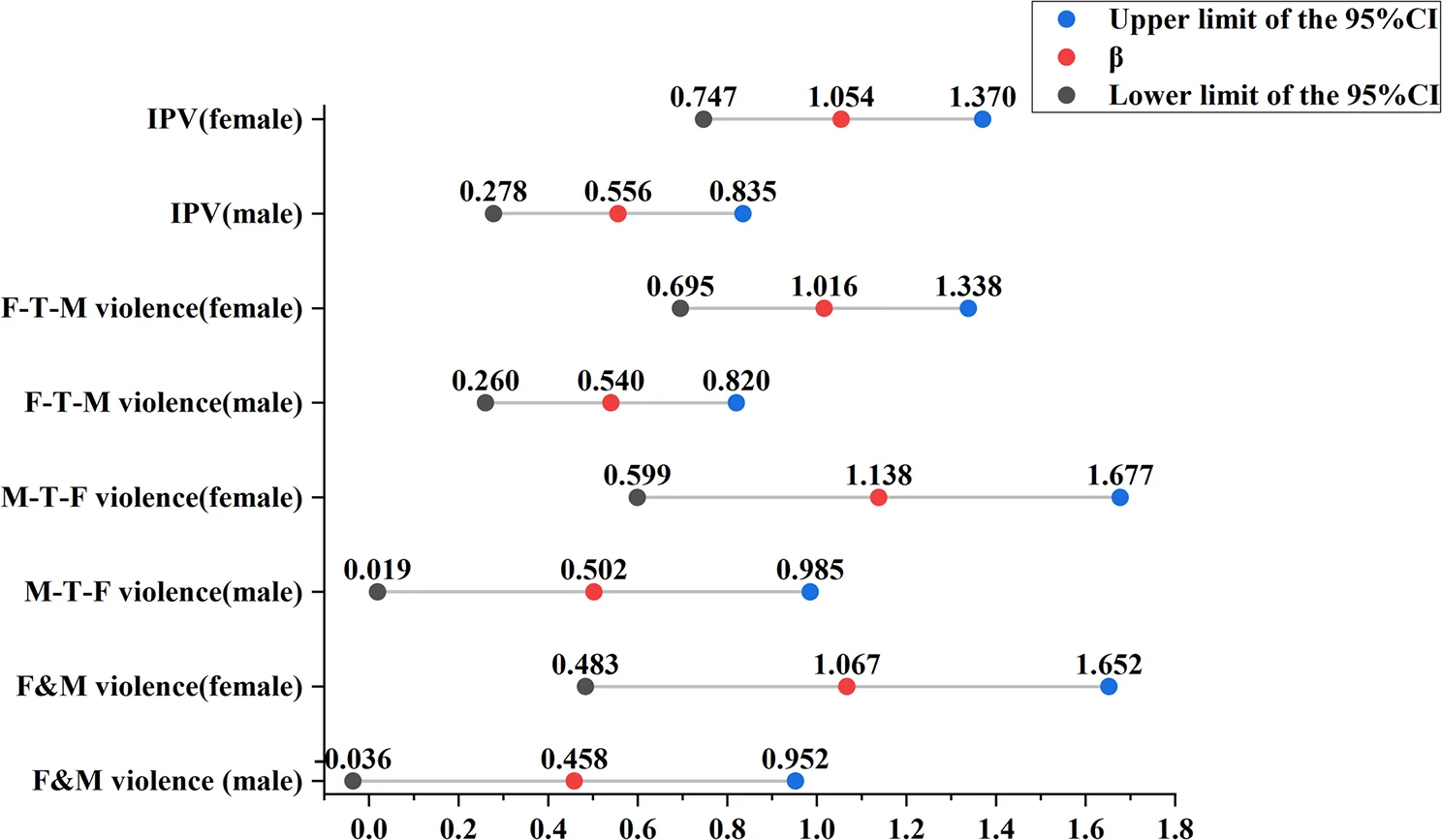
Heterogeneity analysis of the association between IPV and depression by gender. NOTE: Depression was measured as a continuous variable

Compared with females (β = 1.054,95%CI: 0.738, 1.370), the association between IPV and depression was weaker among males (β = 0.556,95%CI: 0.278, 0.835, *P* = 0.017). Compared with females (β = 1.016,95%CI: 0.695, 1.338), the association between father-to-mother violence and depression was weaker among males (β = 0.540,95%CI: 0.260, 0.820, *P* = 0.023). No significant gender difference was found in the association between mother-to-father violence and depression (*P* = 0.106), and no significant gender difference was found in the association between father-and-mother violence and depression (*P* = 0.145). Details are presented in Fig. 3.

## Discussion

To the best of our knowledge, this was the first study to investigate the association and mediating roles between childhood exposure to inter-parental violence and depression in middle-aged and older Chinese adults using 10-year longitudinal follow-up data. This study found that childhood exposure to inter-parental violence was positively associated with depression in middle-aged and older adults. Further analyses revealed that the association between inter-parental violence and depression may operate partly through smoking and drinking. Finally, heterogeneity analysis revealed that the associations of inter-parental violence and father-to-mother violence with depression were weaker in males than in females.

This study first explored the association between childhood exposure to inter-parental violence and depression in middle-aged and older adults, and confirmed a positive correlation between the two variables. One possible interpretation was that inter-parental violence usually occurred in the places considered safest for children, and such violence has been associated with an unhealthy, unstable, and unsettling family environment. When children grow up in stressful environments, their self-esteem and sense of mastery were undermined [17]. Fear for their safety was likely to be related to greater emotional insecurity in children [36]. In addition, parents who experienced more conflict, negativity, frustration, and emotional distress tended to be less sensitive and patient with their children. Declining emotional warmth and rising negative parent-child communication correlated with children’s unhealthy development [37]. Children exposed to high levels of parental conflict may lack positive role models for managing stress, disagreements, and intense emotions [38], which could be associated with an elevated risk of depression in middle-aged and older adults. Additionally, the way children perceive inter-parental violence may also be related to depression [39]. Exposure to inter-parental violence was likely to be distressing for children, who tended to become more anxious about their personal safety and the future of their families; such anxiety has been associated with more severe depressive symptoms [40]. These cognitive patterns may also be linked to depression in middle-aged and older adults [41]. A robustness test using the four-category classification of inter-parental violence yielded results consistent with the main findings.

Having verified the association between childhood exposure to inter-parental violence and depression in middle-aged and older adults, this study further explored the mediating roles of the association. This study found that smoking and drinking played mediating roles in the association between childhood exposure to inter-parental violence and depression in middle-aged and older adults. Drinking exhibited a negative indirect effect, which may represent inconsistent mediation. Inconsistent mediation was a statistical mediation pattern characterized by opposite signs of the indirect effect and the direct effect [42, 43]. This pattern indicated that drinking served as a mediator and masks part of the association between childhood exposure to inter-parental violence and depression in middle-aged and older adults. This may be because, on the one hand, the combination of childhood adversity and hazardous drinking was associated with risk of depression [44]. This finding suggested that interventions targeting problematic drinking among this population may be beneficial, and further supported the potential value of integrating mental health interventions with alcohol-related prevention and treatment efforts [45]. On the other hand, this finding may be related to the measurement method used for drinking. Due to data limitations, the drinking indicator in this study only measured drinking frequency, without accounting for drinking volume, heavy drinking, problem drinking, or former drinking. Future research should systematically examine the associations between multi-dimensional drinking behaviors and depression. This study found that smoking mediated the association between inter-parental violence and depression, which may be related to the mediating role of lifestyle in the association between early adverse experiences and subsequent mental health status [46].

To thoroughly unpack population disparities in the association between childhood exposure to inter-parental violence and depression in middle-aged and older adults, this study further explored gender differences in this association. The findings revealed that father-to-mother violence, mother-to-father violence, and father-and-mother violence were all positively associated with depression. The study also found that the associations between inter-parental violence and depression, and between father-to-mother violence and depression were stronger among female respondents, which was consistent with previous research [47]. The association between family conflicts and difficulties in psychological adjustment tended to be stronger in females [48]. Given that males and females may adopt distinct coping strategies [49], such differences should be taken into account when developing health intervention measures.

### Strengths

This study has several strengths. First, to the best of our knowledge, it was the first study to examine the association between childhood exposure to inter-parental violence and depression in middle-aged and older adults in China, providing empirical evidence related to adolescent development. Second, this study found mediating factors in the association between inter-parental violence and depression, highlighting the importance of lifestyle in alleviating depression among middle-aged and older adults. Third, this study investigated the gender heterogeneity of the association between childhood exposure to inter-parental violence and depression in middle-aged and older adults, which may contribute to a better understanding of parent-child relationship dynamics.

### Limitations

However, this study has some limitations. First, data on childhood exposure to inter-parental violence was based on the current recollections of middle-aged and older adults, which may be susceptible to recall bias. Depression in this study was assessed based on scale scores; future studies could adopt more rigorous clinical diagnostic criteria for verification. In this study, missing demographic data were supplemented or calculated across waves for the same respondent. The assumption underlying this approach was that these variables remain stable or change in a predictable manner. Although this strategy reduced sample attrition, it may have masked true individual-level changes and introduced measurement error or bias. Second, this study only demonstrated correlational associations rather than causal relationships. This study adopted lagged mediation analysis, the lagged structure improved temporal ordering but did not fully establish causal mediation. Third, inter-parental violence encompassed multiple dimensions, and this study only examined physical violence. Future research should adopt more comprehensive perspectives to examine inter-parental violence. Among them, the sample size of the group only exposed to mother-to-father violence was small, which limited the statistical precision for this subgroup. Future studies should recruit larger samples for this specific group. In addition, due to data limitations, the drinking measure used in this study only distinguishes up to “drinking more than once a month”. In mediation analysis, the observed findings may correlate with residual confounding factors, healthy-user bias, social participation effects, or the use of three-category coding for the drinking variable instead of quantity, more detailed frequency, or problem drinking.

## Conclusion

Using data from the CHARLS 2011–2020, this study found that childhood exposure to inter-parental violence was positively associated and depression in middle-aged and older adults. Additional research revealed that the association between inter-parental violence and depression may operate partly through smoking and drinking. This finding had implications for lifestyle interventions for survivors of childhood exposure to inter-parental violence. Finally, this study discovered that the associations between inter-parental violence and depression, and between father-to-mother violence and depression, were stronger among female respondents. Generally, it was necessary to focus on depression among individuals who were exposed to inter-parental violence in childhood.

## Data Availability

This data were derived from the CHARLS. They are opened to everyone. Researchers who want to use these data can visit http://charls.pku.edu.cn/.

## Abbreviations

CHARLS: China Health and Retirement Longitudinal Study
CES-D-10: Center for Epidemiologic Studies Depression Scale-10
IPV: Inter-parental violence
F-T-M: Father-to-mother
M-T-F: Mother-to-father
F&M: Father-and-mother
CI: Confidence interval
